# Mechanistic Insights Into Probiotic Properties of Lactic Acid Bacteria Associated With Ethnic Fermented Dairy Products

**DOI:** 10.3389/fmicb.2019.00502

**Published:** 2019-03-26

**Authors:** Tamoghna Ghosh, Arun Beniwal, Anupama Semwal, Naveen Kumar Navani

**Affiliations:** Chemical Biology Lab, Department of Biotechnology, Indian Institute of Technology Roorkee, Roorkee, India

**Keywords:** probiotics, ethnic fermented food, *Lactobacillus*, dairy, cancer, fermentation

## Abstract

Gut microbes and their metabolites maintain the health and homeostasis of the host by communicating with the host *via* various biochemical and physical factors. Changing lifestyle, chronic intake of foods rich in refined carbohydrates and fats have caused intestinal dysbiosis and other lifestyle-based diseases. Thus, supplementation with probiotics has gained popularity as biotherapies for improving gut health and treating disorders. Research shows that probiotic organisms enhance gastrointestinal health, immunomodulation, generation of essential micronutrients, and prevention of cancer. Ethnically fermented milk and dairy products are hotspots for novel probiotic organisms and bioactive compounds. These ethnic fermented foods have been traditionally prepared by indigenous populations, and have preserved unique microflora for ages. To apply these unique microflora for amelioration of human health, it is important that probiotic properties of the bacterial species are well studied. Majority of the published research and reviews focus on the probiotic organisms and their properties, fermented food products, isolation techniques, and animal studies with their health pathologies. As a consequence, there is a dearth of information about the underlying molecular mechanism behind probiotics associated with ethnically prepared dairy foods. This review is targeted at stimulating research on understanding these mechanisms of bacterial species and beneficial attributes of ethnically fermented dairy products.

## Introduction

A century ago, Élie Metchnikoff observed the beneficial role of fermented milk on the health of Bulgarian peasants and postulated the positive effect of selected microbiota on gastrointestinal health of the villagers due to the daily intake of soured or fermented milk (Kaufmann, 2008). A century later, this observation has led to a revolutionary concept of treating gastrointestinal diseases associated with dysbiosis with gut microbiota. Although, modern researchers are currently exploiting the idea by introducing functional foods (nutraceuticals) and fecal transplantations, yet indigenous communities unaware of the underlying principle have been naturally preparing and consuming fermented milk and milk products since the domestication of livestock species. Through fermented products, ethnic communities have been conserving unique microbial diversity historically. Lactic acid bacteria have been employed to ferment milk over 6,000 years ago, mostly when Babylonian, Egyptian, and Indus valley civilization flourished (Fox, 1993; Robinson et al., 2006). Traditional fermented foods are those which are being consumed for centuries, predating historical evidences, and proved essential for the welfare of the community (Hesseltine, 1965). The process of milk fermentation was a natural phenomenon, as lactic acid-producing microorganism in milk could normally ferment and acidify milk. The early tribal people noticed that with incubation of the organisms, they could preserve food for a longer time with increased flavor, texture, and aroma (Mayo et al., 2010). Thus, a long tradition of handling and storing food with certain microbes was handed down from generation to generation in communities and tribes (Caplice and Fitzgerald, 1999).

Early people in tropical countries were the heavy consumers of fermented milk products, whereas, people from North America and Europe were hefty consumers of milk (Mayo et al., 2010). Currently, about 20–30% of the foods consumed are fermented products. Milk being a rich source of nutrition produced by almost all domesticated mammals in every part of the world identifies itself as a popular culture for all kinds of societies. Buffalo and cow milk are ubiquitous, whereas, yak and camel milk and its products were consumed by ethnic and tribal people of Himalaya and mid-west Asia and Northern Africa respectively (Mayo et al., 2010; Tamang et al., 2012, 2016a,b,c). Milk in the different regions comprises of different levels of micronutrients, proteins, sugars, and fats, depending upon conditions of the region and producer animals, which subsequently alter the characteristics of the microorganisms and their functions. Due to changing lifestyle and globalization, there are alterations in preparation for some of the fermented dairy products as compared to the ethnic preparations; however, the microorganisms are nearly conserved. These fermented milk products preserve the nutritional value through the development of lactic acid, acetic acid, flavor, enhancement of essential amino acids, metabolites, essential fatty acids, and detoxification of undesirable metabolites. In 1965, Lilley and Stillwell termed these microorganisms as “probiotics” and demonstrated that the substances secreted by these organisms could stimulate the growth of other microorganisms and hosts (Fuller, 1992).

Probiotics isolated from traditional dairy products comprise species of lactic acid bacteria with a history of safe use. The employment of live bacteria incorporated in food is included in the European Qualified Presumption of Safety list (Leyva Salas et al., 2018). Many probiotic lactic acid bacterial strains isolated from different dairy products have also undergone review and testing and have fulfilled the FDA “GRAS” status for use in fermented dairy product and other food items. Even though, probiotic properties have been studied and used safely for decades, yet the absence of molecular details about the mechanism of their healthy attributes (cause-and-effect relationship) concerns the regulatory authorities for their safe usage. Thus, an understanding of the mechanisms will enable therapeutic usage of scientifically supported probiotic supplements with well-defined health claims in desired fermented products. To get an insight into the mechanisms, a number of probiotic lactic acid bacteria have been isolated either from the ethnic fermented dairy products or native microbiota of healthy individuals. It has been observed that all the probiotic bacteria do not share all the attributes. Specific strain depicts precise function which is not observed in other strains of same species. However, it has also been observed that not all the probiotics are unique and many of them share some common functions (Sanders et al., 2018). The role of probiotics in maintaining the health of the human gut has been mostly documented (Butel, 2014; Vandenplas et al., 2015). Using probiotics and metabolites as gut modulators, the function and composition of the bacterial community can be selectively altered. To improve the role of probiotics isolated from an ethnic fermented dairy product in stimulating human health, it is crucial to study their mode of action. This review summarizes some of the underlying molecular mechanisms behind different attributes such as, gastrointestinal health, immune modulation, cholesterol reduction, cancer mitigation, and production of bioactive metabolites by microbiota associated with indigenous fermented milk products.

## Traditional Dairy Products and Microbiota Associated with the Products

Fermented milk products also known as cultured dairy products are dairy foods which have been fermented by a consortium of lactic acid bacteria (LAB) responsible for the curdling or souring of milk (FAO/WHO, 2003). LAB are best-suited organisms for milk fermentation while preserving the taste and nutritional properties. These bacteria are non-sporulated, mostly anaerobic in nature, and are only capable of growing in rich nutritional conditions that provide growth factors like vitamins, amino acids, and nucleotides. Bacterial members associated with fermented dairy products belong to the genera of *Lactobacillus*, *Lactococcus*, *Leuconostoc*, *Pediococcus*, *Bacillus*, *Propionibacterium*, and *Bifidobacterium*. These bacteria live in same ecological niches and act mutualistically. There are approximately 400 traditional and fermented milk products comprising a diverse group of microorganisms giving rise to different sensory properties. There are two different classes of milk products based on fermentation:

Class I: Bacterial lactic acid fermentation: (1) Fermentation by mesophilic bacteria (acidified milk, buttermilk, filmjolk, and langfil), (2) fermentation by thermophilic and mesophilic bacteria (yoghurt, dahi, Bulgarian buttermilk, zabadi).

Class II: Fungal and bacterial lactic acid fermentation: Fermentation by bacteria as well as fungi. E.g. alcoholic milk (Acidophilus yeast milk, Koumiss, and kefir) and Moldy milk (Villi).

The varieties of milk products depend upon different types of milk and starter cultures, sugars, aromatic compounds, and grains. These varieties are developed using primary starter cultures (which participate in primary acidification) and secondary starter cultures (which participate in generating aroma, flavor, and texture). Genera used in primary culture are *Lactobacillus* sp., *Leuconostoc* sp., *Streptococcus* sp. (Parente and Cogan, 2004), whereas, the genera associated with secondary starter cultures are *Propionibacterium* sp., *Brevibacterium* sp., *Debaryomyces* sp., *Geotrichum* sp., *Penicillium* sp., and *Enterococcus* sp.

Characteristics of naturally fermented milk depend upon the availability of the milk in respective regions. However, fermented milk like Zeer, Kad, Zabady, Laban, Rayeb, and Shubat from Northern Africa, Morocco, and mid-west Asian countries; Ergo from Ethiopia; Amasi from Zimbabwe; Roub from Sudan; Chhurpi, Mohi, Philu, Somar, and Shoyu from Himalayan region; and flmjölk and långfl from Sweden have same characteristics of fermentation. These products are majorly dominated by mesophilic lactic acid bacteria, which lower pH, improve sensory properties, inhibit other bacterial spoilage, and improve health (Table 1). In primary fermentation, *Lactococcus lactis* and *Lactococcus lactis* subsp. *cremoris* are found to be most dominating ones. Other important bacteria commonly found in these products are *Lactobacillus plantarum*, *Lactobacillus casei*, *Lactobacillus paracasei*, *Leuconostoc* sp., *Enterococcus* sp., and *Pediococcus* sp. In tropical countries, *Lactobacillus* sp. like *Lactobacillus helveticus*, *Lactobacillus fermentum*, *Lactobacillus acidophilus*, and *Lactobacillus brevis* are also prevalent (Gonfa et al., 2001; Mathara et al., 2004; Patrignani et al., 2006). In raw milk, a very high number of yeast species are also found (Gadaga et al., 2000; Gonfa et al., 2001; Benkerroum and Tamime, 2004). They enhance the flavor and texture of milk products. Major yeast species found are *Candida lusitaniae*, *Saccharomyces cerevisiae*, and *Kluyveromyces marxianus* (Gadaga et al., 2000; Benkerroum and Tamime, 2004). Some of the milk products are partly dried like Leben, Zeer, and Than; while some are maintained in oil like Shanklish. Few are mixed with spices like Mish and some are mixed with wheat and cereals like Kishk and Kadhi. Salted cheeses like Feta, Lighvan, and Domiati are heavily consumed in Middle Eastern Asia and Balkans, which also represent air-dried and sundried cheeses from Northern Africa (Kosikowski and Mistry, 1997). Many of the North African and middle western Asian fermented products are made up of camel milk like Chal, Unda, Shubat, and Susa. A rich diversity of traditional fermented milk products is present in the Himalayan region, mainly fermented from yak, buffalo, and cow milk. Chilika curd is one of the ethnic fermented foods with an exceptionally extended shelf life that is prepared by ethnic community of Chilika in Odisha state of South-Eastern India. Chilika is made up of special cup made up of bamboo basket using milk of Chilika Buffalo (Nanda et al., 2013). The lactic acid bacteria present in Chilika curd have been observed to exhibit higher antifungal activity due to the presence of compounds such as 3-hydroxy fatty acid, caproic acid, and fungicins. Nunu, a fermented milk consumed in Ghana and western part of Africa, is known to harbor strains of *Lactobacillus*, *Leuconostoc*, *Enterococcus*, *Weissella*, and *Pediococcus spp.* with health beneficial properties (Akabanda et al., 2013). Fermented milk products also represent an important part in the staple diet for countries like Afghanistan, Pakistan, India, Nepal, Bhutan, China, and Myanmar. Some of the indigenous fermented products are Dahi, Chhurpi, Churkam, Chhu, Somar, Mohi, Philu, Maa, and Shoyow. Some of the products are ubiquitous to the Indian subcontinent like Dahi (yoghurt), Mohi (buttermilk), whereas products like Chhu, Churpi, and Somar are restricted to inhabitants of Himalayan foothills where yaks are reared (Dewan and Tamang, 2006; Tamang and Samuel, 2010; Rai et al., 2016). Some naturally fermented milk products found in Himalayan regions were prepared from the old technique known as back-slopping and it is still used to preserve the microflora present in these fermented products. Such products include ethnic fermented products of Bhutan such as dahi, datshi, hard-chhurpi (churkam/chugo) mohi, gheu, and hitpa (Shangpliang et al., 2017). The traditional back-slopping in dairy fermentation is different from mono-culture fermentation in enhancing the probiotic characteristics as these contain wild-type strains with enriched biosynthetic capacity, higher genetic diversity, and enhanced ability to produce antimicrobials such as bacteriocins. Presence of a higher number of bacilli in contrast to cocci in the Himalayan fermented milk products implies that the milk in different regions supports a set of consortium for their particular characteristic fermentation and qualities (Dewan and Tamang, 2007). The dominant species in the Himalayan fermented milk products are *L. plantarum*, *Lactobacillus bifermentans*, *L. lactis* subsp. *cremoris*, *L. paracasei*, *L. alimentarius*, *L. kefir*, *L. bulgaricus*, and *Enterococcus faecium* (Dewan and Tamang, 2007). Indigenous fermented foods have been prepared and consumed for thousands of years and maintain the natural microflora present in them. The variety of microorganisms present in these fermented food are able to create flavors that are difficult to imitate in commercial products where pure starter cultures are used for preparing them (Thapa and Tamang, 2015). Since isolation and identification of these bacteria are based on culture-dependent methods, it is difficult to comprehend the true landscapes of their diversities and benefits. Molecular techniques such as denaturing gradient gel electrophoresis have revealed the presence of *Leuconostoc mesenteroides, Lactobacillus helveticus, L. kefiranofaciens, L. lactis, L. kefir*, *and L. casei* as the dominant microorganisms present in Tibetan kefir (Zhou et al., 2009). In another study carried out on the diversity of Mongolian traditional fermented dairy products using pyro-sequencing, it was found that there was a correlation between animal species and the genus *Lactobacillus* which was found to be the core foundation in Mongolian fermented milks. *L. kefiranofaciens, L. helveticus,* and *L. delbrueckii* were the predominant species sequenced using NGS for ethnic Khoormog, Airag, and Tarag fermented samples, respectively (Oki et al., 2014).

**Table 1:** Therapeutic and beneficial properties of ethnically fermented dairy products and associated microorganisms.

Traditional fermented dairy foods	Microbial flora	Associated actions	References
Koumiss	*L. casei* Zhang (LCZ) *Lactobacillus* sp.	Increased host immunity in gut by systemic immune response by secretion of IL-12, IFN-γ, sIgA, IL-10, and reduced level of pro-inflammatory cytokines (IL-1). Suppressed effect on pathogens such as *Acinetobacter* and *Pseudomonas*. Increased level of short-chain fatty acids (SCFA) Helped in cholesterol assimilation. Enhanced synthesis of ACE inhibitors and GABA.	Ya et al., 2008; Dong et al., 2015 Wang et al., 2014; Hor et al., 2018; Guo et al., 2015 Sun et al., 2009
Kefir	*Lactobacillus kefir, Lactobacillus kefiranofaciens, and Lactobacillus kefirgranum* *L. plantarum* MA2 *L. kefiri* D17, *L. plantarum* B23 and *L. acidophilus* LA15	Showed antibacterial activity by production of bacteriocin Reduced inflammation in epithelial cells of intestine Reduced cholesterol level in serum. Produced an EPS known as kefiran. Reduced cholesterol, LDL, and triglyceride in male Sprague–Dawley (SD) rats Induced apoptosis of Caco-2 and HT-29 cancer cells and decreased transforming growth factor (TNF-α and TNF-β) in HT-29 Displayed anti-proliferative effect in different cancer cell lines	Luo et al., 2011 Seo et al., 2018 Wang et al., 2008 Bonczar et al., 2016 Huang et al., 2013 Zheng et al., 2013 Khoury et al., 2014 Furukawa et al., 2000 Chen et al., 2007
Katak	*L. brevis*	Showed antifungal activity against *Aspergillus* and *Penicillium* sp.	Tropcheva et al., 2014
Dahi	*L. rhamnosus* S1K3 *L. acidophilus*	Produced antimicrobial compounds to resist foodborne pathogens. Enhanced integrity of tight junction protein by up-regulating claudin 1 gene. Increased expression of human β-Defensin-2 and β-Defensin-3. Induced the expression level of IL-4, Toll-like receptor (TLR) at Peyer’s patches and IgA level in serum Produced EPS Enhanced riboflavin production	Kemgang et al., 2016 Vijayendra et al., 2008 Jayashree et al., 2010
Camel milk fermented products	*L. plantarum*, *L. acidophillus* and *L. reuteri* and *L. lactis*	Produced EPS Displayed anti-proliferation of MCF-7, Caco-2 and HeLa cells. Production of ACE inhibitors	Abushelaibi et al., 2017 Ayyash et al., 2018
Tibetan Kefir	*L. plantarum* YW11 *Butyricoccus* sp. and *Blautia* sp. *L. plantarum* Lp27	Produced of EPS and elevated level of superoxide dismutase, catalase, glutathione peroxidase to protect from oxidative damage. Decreased level of malondialdehyde Decrease serum total cholesterol, LDL-cholesterol, and triglycerides in hypercholesterolemic SD rats	Zhang et al., 2017 Huang et al., 2013
Iranian dairy product	*L. brevis*	Assimilated cholesterol	Iranmanesh et al., 2014
Tulum Cheese	*L. fermentum*	Assimilated cholesterol	Tulumoğlu et al., 2014
Khadi	*P. pentosaceus* GS4	Showed anti-proliferation activity in HCT-16 mammalian cells, increased expression of pro-apoptotic molecules NF-κB and p-Akt and produced conjugated linoleic acid	Dubey et al., 2016
Tarkineh, Shiraz	*L. plantarum* and *L. lactis subsp*. lactis *Kluyveromyces marxianus* AS41	Induced of apoptois and produced anticancer peptides Showed anti-proliferative activity on cancer cells. Down-regulated Bcl-2 expression and up-regulated BAD expression	Haghshenas et al., 2015 Saber et al., 2017
Swiss Cheese	*Lactobacillus helveticus* R389	Enhanced immune system by increasing IgA and CD4 positive cells. Decreased IL-6 and increased IL-10 expression.	De LeBlanc et al., 2005
Kajmak	*L. mesenteroides*, *L. lactis* and *L. paracasei*	Enhanced flavor by production of diacetyl, acetate, and ethanol	Jokovic et al., 2008
Rabadi	*L. plantarum* RYPR1	Exhibited hypocholesterolemic effect due to bile salt hydrolase activity	Yadav et al., 2016
Brazilian Kefir	*L. lactis* subsp. cremoris MRS47	Modulated lipid profile by generation of SCFA	Vieira et al., 2017
Italian Cheese	*L. helveticus* PR4	Produced ACE inhibitors, antibacterial peptides and GABA	Minervini et al., 2003; Siragusa et al., 2007
Chhurpi (Yak cheese)	*L. fermentum*	Produced phytase, exhibited dephytanation in finger millets and Durum wheat under *in vitro* gastrointestinal conditions	Sharma et al., 2018

Recently, metagenomic investigations of the naturally ethnic fermented milk products such as churkam, churpi, mar, and dahi have shown that Proteobacteria (*Acetobacteraceae*) and Firmicutes (*Streptococcaceae, Lactobacillaceae*) were the two most predominant members of the microbial communities in these traditional fermented products. *L. helveticus* and *L. lactis* were the predominant lactic acid bacteria while *Gluconobacter and Acetobacter* spp. were the predominant acetic acid bacteria present in these fermented products (Shangpliang et al., 2018). Therefore, these metagenomic and culturonomic approaches will provide a wider spectrum of microbes associated with these indigenous products, along with a better perspective of their attributes contributing toward the welfare of human being.

## Molecular Perspective of Ethnic Dairy Products in Gastrointestinal Health

**Figure 1:**
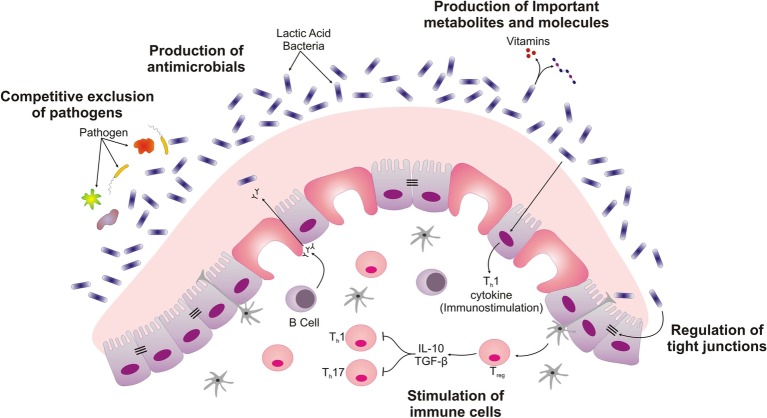
Schematic displaying different ways by which probiotics exert their beneficial roles in intestine. LAB isolated from ethnic dairy product are responsible for competitive exclusion of pathogens, secretion of important metabolites and molecules such as bacteriocins. These Probiotics create mucus barrier by stimulating the Goblet cells. The interaction of *Lactobacillus* with intestinal epithelial cells also differentiates immune cell and regulates the barrier function of intestinal epithelial cell.

The Human gut microbiome consists of all the living microorganisms existing in association with the gut of human. These living microorganisms include bacteria, archaea, eukaryotes, and virus. These gastrointestinal microbes present in a continuum with ingested bacteria maintain metabolic homeostasis. Moreover, the single layer of specialized epithelial cells present in the intestine form a highly complex structured network which is the major intestinal defense system present in gut against pathogens and designated as intestinal barrier function. The epithelium of gut uses different defense mechanisms against the microbiota such as immune response (innate or acquired), mucus layer secretion, as well as integrity and turnover of the epithelial cell (Bron et al., 2012). In order to maintain the intestinal barrier function, adjacent epithelial cells of the gut form tight junctions with each other. These junctions act as a barrier that is impermeable to particulate things and liquid materials. Other cells of epithelium i.e. Goblet and Paneth cells also support barrier function, thereby contributing as a part of innate immune system. Collectively, all these barriers decrease the load of pathogens at the interface between epithelium and lumen. However, various intestinal linked inflammatory diseases such as inflammatory bowel disease (IBD) have been manifested by a leaky intestinal barrier. Transient passage of probiotic bacteria in upper gastrointestinal tract (GIT) at a concentration higher than 10^7^ bacteria overwhelms the normal population of microbiota. These probiotic bacteria, therefore, possess a higher access to the microvilli, mucosa, and other cells of the immune system. A number of researchers have found that probiotics can activate both the innate and adaptive immune systems, and thus provide better protection against pathogens (McFarland, 2006; Sánchez et al., 2017). In different studies, consumption of ethnic probiotic bacteria in dairy product and their interaction with intestinal cells initiate an immunological response. The surface marker present on probiotic bacteria such as exopolysaccharides, lipoteichoic acid, fibronectin-binding proteins, and mucus-binding proteins are key factors responsible for crosstalk with the host intestinal epithelium cells (Sanders et al., 2018). These interactions are important because they influence the production of chemokines and cytokines that are secreted by intestinal enterocytic cells (Figure 1; Gill and Prasad, 2008). For example, in a study conducted on Mongolian Koumiss, a potent *L. casei* Zhang (LCZ) strain was isolated after screening 240 isolates of *Lactobacillus* sp. This strain has proven to have high binding affinity toward intestinal Caco-2 cell lines. Further role of LCZ probiotic in maintaining host immunity homeostasis was observed using *in vivo* experiments where it was observed that the LCZ induced gut immune response by secretory IgA (sIgA) secretion in intestine and also undergoes systemic immune response by secretion of IL-12 and IFN-γ in mice model (Ya et al., 2008; Dong et al., 2015). In a clinical study on this strain, it has been found that LCZ carried an ability to modulate the composition of fecal microbiota in both elderly and adult subjects. The strain exhibited growth-suppressive effect on pathogens such as *Acinetobacter* and *Pseudomonas*. Furthermore, it was also observed that there was an increase in the level of short-chain fatty acids (SCFA) for a prolonged period in the intestine (Wang et al., 2014). A very recent 12-month randomized clinical trial using this same strain was carried out on Malaysian populations where the study showed that LCZ strain alleviated gastrointestinal disorders and upper respiratory tract infections in the full-fledged population. It was observed the strain activated both B and T cells, and increased the level of anti-inflammatory cytokines such as IL-10 and reduced the level of pro-inflammatory cytokines (IL-1) (Hor et al., 2018). Regulatory T cells (T_reg_) constitute a key source of anti-inflammatory cytokine IL-10, and are further involved in maintenance of immune tolerance and regulation of appropriate immune response mediated by T cells. Recent, randomized, double-blind clinical trial using *L. plantarum* showed that there was significant lowering of sepsis and lower respiratory tract infections among infants in rural India. The findings suggest that the probiotics LAB can effectively prevent a large proportion of neonatal sepsis in developing countries (Panigrahi et al., 2017).

Other modes of action of probiotics include competitive exclusion of the pathogens. The antimicrobial properties associated with probiotic kefir were reported on various pathogens. The production of bacteriocin and lactic acid by the probiotic *Lactobacillus* present in the kefir has been found to inhibit *Bacillus subtilis, Staphylococcus aureus*, *Escherichia coli*, and *Pseudomonas aeruginosa* (Luo et al., 2011). However, kefir-derived yeast probiotics have also been reported to produce organic acid, hyaluronic acid, acetic acid, and ethanol depending on the prevalent probiotic strain present during fermentation. The pathogens and probiotics behave in different manner to these metabolites. Dairy yeast strains generally respond to ethanol stress by reprogramming their metabolism and survive during various stresses of fermentation condition in a better form as compared with the pathogens (Lara-Hidalgo et al., 2017; Saini et al., 2017a, 2018). Another ethnic, curd-like product of Bulgaria called Katak has also been used traditionally for centuries using eve’s milk. Katak has proven as a promising candidate for isolation of bacteria showing antibacterial and antifungal activity. *L. brevis* have been isolated from Katak which have the ability to suppress the growth of *Aspergillus* and *Penicillium* sp. (Tropcheva et al., 2014). Only a few studies conducted on probiotic bacteria derived from ethnic fermented dairy products have addressed human response to *Lactobacillus* at the molecular level. *L. rhamnosus* HN001 isolated from dairy products has been found to improve the immune system against multiple pathogens. Recently, in phase II clinical trial, *L. rhamnosus* was able to decolonize *S. aureus* in geriatric patients. It was concluded that, probiotic strain *L. rhamnosus* outcompetes pathogens for important resources, thereby preventing colonization of pathogenic strains. *L. rhamnosus* HN001 was able to stimulate and help immune system in preventing colonization of *S. aureus* (Eggers et al., 2018). In a randomized clinical trial, consumption of probiotic cheese containing *L. rhamnosus* HN001 and *L. acidophilus* by elderly volunteers changed intestinal microbiota, lowered the count of *Clostridium difficile*, and increased IgA concentration compared to the cheese consumed without probiotics.

In human intestinal epithelium, the interaction between adjacent cells and cell-basement membranes form a crucial barrier that prevents the translocation of the microbes to the sub-epithelial layer. This adherence is governed by tight junction, gap junction, adherence junction, and desmosomes. The mechanistic role of probiotics reported in various studies associated with the strengthening of mucosal barrier function is mainly directed toward examining the ability of probiotic bacteria to prevent alterations in bridging proteins and tight junction present in the epithelial cell by various *in vitro* and *in vivo* models. A study conducted on *L. rhamnosus* S1K3 isolated from local Indian fermented milk product, dahi, showed that consumption of the strain for a period of 30 days could reduce *Salmonella enterica* due to the production of antimicrobial compounds. In this study, it was also observed that the pathogen was responsible for disruption of the tight junction proteins by down-regulating the expression of claudin 1 gene. Interestingly, when the probiotic strain S1K3 was administrated, it was again able to strengthen the integrity of the tight junction protein by up-regulating claudin 1 gene. There are two major antimicrobial peptides produced by intestinal epithelial cells in intestinal fluid, termed as defensins and cathelicidins. In the same study, probiotic bacteria induced an increase in the expression of human β-Defensin-2 and β-Defensin-3. The strain was able to induce the expression level of IL-4, Toll-like receptor (TLR) at Peyer’s patches, and IgA level in serum and intestinal fluid (Kemgang et al., 2016). These factors present in the probiotic-fed group are collectively responsible for lowering down the level of pathogenic *Salmonella enterica* in feces. Therefore, probiotic strains depict enhanced intestinal barrier function by the production of the antimicrobial peptide, increased production of tight junction protein, stimulated intestinal mucosa, increased IgA responses, and prevented epithelial cell apoptosis.

Few studies have shown that probiotic *Lactobacilli* isolated from ethnic fermented product possess the ability to modulate gut residential population as a way to treat diseases like irritable bowel syndrome (IBS) and IBD. IBD includes both the ulcerative colitis (UC) and Crohn’s diseases. These diseases occur in the area where the microbes (e.g. *Helicobacter hepaticus*) are prevalent in higher numbers. Microbiota present in a normal healthy individual differs from that of the person who is suffering from IBD. Firmicutes and *Bacteroides* are the predominant residential communities which are present in healthy individuals, whereas during dysbiosis of the gut in IBD patients, altered levels of predominant community and expansion in the level of Enterobacteriaceae family have been observed. Higher diversity of microbes present in normal individual provides colonization resistance against pathogens. The overall response of the normal residential microbes of the intestine is immunological tolerance and homeostasis. In contrast, during dysbiosis, the altered microbes activate deregulated T_h_17 and T_h_1 effector cells and in turn mediate inflammation of the intestine. The inflammation further leads to the formation of UC and Crohn’s diseases in genetically susceptible host. The modulation of intestinal gut bacteria through the fermented dairy food containing probiotic bacteria offers a promising strategy to alleviate IBD diseases. A number of microbial strains, especially from the genera of *Lactobacillus, Bifidobacterium,* and *Faecalibacterium*, protect the host from mucosal inflammation by a number of different mechanisms which include stimulation of anti-inflammatory cytokine IL-10 and down-regulation of inflammatory cytokines (Llopis et al., 2008). IL-10 has been found to stimulate T_h_2 cells and is considered as a typical marker for anti-inflammatory effects. TNF and IL-12 are pro-inflammatory markers associated with stimulation of T_h_1 cell and induction of IFN-γ by T-cell. An *in vivo* study conducted on colitis mice model fed with probiotic *L. plantarum* showed an increased anti-inflammatory effect on micro-integral membrane protein (MIMP), gut barrier, and inflammatory cytokines. These MIMPs may further act as target of clinical therapy for IBD patients (Yin et al., 2018). An *in vivo* study on extracellular vesicle (EV) of three *Lactobacillus* sp. (*Lactobacillus kefir, Lactobacillus kefiranofaciens*, and *Lactobacillus kefirgranum*) exhibited significant reduction of inflammation in epithelial cells of intestine. Administration of EV into IBD-induced mice models alle*via*ted rectal bleeding and weight loss, and increased stool consistency. Earlier, IL-8 has been reported as a crucial factor in stimulating the inflammation-based pathogenesis in IBD. It tends to be produced from the epithelial mucus lining in patients suffering from IBD. This study also reported that EV of *Lactobacillus* inhibits the expression of IL-8 in the intestine (Seo et al., 2018).

Furthermore, probiotic microorganisms and other commensals present in the gut can build a tolerant state which is mediated by the action of Toll-like receptors (TLR). Sensing of probiotics by dendritic cells (DCs), epithelial cells, and macrophages is governed by the TLR receptors which work as pattern recognition receptors (PRRs). Activation of these receptors induces pathways that trigger adaptive immune cells and pro-inflammatory T_h_17 and T_h_1 helper cells. It has been observed that TLR9 signaling is critical to facilitate anti-inflammatory effect of probiotics. However, there are studies where TLRs, such as TLR3 and TLR7, have also been implicated in the tolerance induced by probiotic cells. After activation by probiotic bacteria, DCs initiate differentiation of T_h_0 to T_reg_, which has been observed to exhibit an inhibitory effect on T_h_1 and T_h_17 inflammatory responses. The probiotics have been found to suppress the intestinal inflammation *via* down-regulation of TLR expression, decreased secretion of TNF, and inhibition of NF-κB signaling pathway. It has been observed that different *Lactobacillus* strains have ability to elicit a variable response in terms of cytokine production in different immune cells. For example, Van-Hemert et al. (2010), while working on different strains of *L. plantarum*, isolated from 48 different sources, observed induction of different concentrations of IL-10 and IL-12 during immune modulation. Future clinical trials guided by these parameters will provide further insights into the exact role of individual probiotic strain in immune modulation. Looking forward, there might be a need of focused selection and smarter manipulation of gut microbiota with the best strain of probiotics (Van-Hemert et al., 2010).

The presence of EPS in LAB allows the surface molecules to interact with host and protects probiotics from the harsh gut environment as demonstrated from various studies on LAB isolated from dairy products. EPS produced by dairy LAB has been observed to provide various physiological benefits such as induction of cytokines (IFNγ and IL-1), antitumor activity, macrophage activation, cholesterol reduction ability, and also enhanced colonization of the probiotics in gastrointestinal tract (Kitazawa et al., 1998, 2000; Pigeon et al., 2002; Chen and Chen, 2013). *L. kefiranofaciens* isolated from ethnic kefir grains produces an exopolysaccharide known as kefiran. Other isolates like *Lactobacillus* sp. KPB-167B, *L. kefiranofaciens*, *and Lactobacillus kefir* had been also described to produce EPS (Wang et al., 2008). In other studies, exopolysaccharide-producing probiotics, *L. lactis*, *and L. plantarum* were also isolated from camel milk, dahi, and other ethnic dairy products (Vijayendra et al., 2008; Abushelaibi et al., 2017). In a study by Zhang et al. (2017), the effect of EPS produced by *L. plantarum* YW11 isolated from ethnic Tibetan kefir on the gut microbiota and oxidative stress in an aging mouse model was investigated. A dose of 50 mg/kg per day was able to relieve oxidative stress in mice by increasing the level of superoxide dismutase, catalase, and glutathione peroxidase, and decreasing the level of malondialdehyde. EPS was also able to modulate the gut microbiota selectively by increasing the abundance of *Butyricicoccus* sp. and *Blautia* sp. and led to enhanced secretion of SCFA (Zhang et al., 2017).

Currently, researchers are making effort to exploit the benefits of gut microbiota by replicating the natural milieu of intestine. Fecal microbiota transplantation (FMT) is one such promising therapy which is gaining acceptance for treating autoimmune and infectious diseases. Ulcerative colitis and *Clostridium difficile* infection have been successfully treated using FMT; however, issues regarding the presence of unknown components still cast uncertainty about the safety of this approach (Gupta et al., 2016). Mostly, microbial strains present in FMT responsible for therapeutic effect are still unknown. Some bacterial taxa associated with FMT are *Bacteroides*, *Bifidobacteria*, *Clostridial* clusters, and *Lactobacilli* (Gupta et al., 2016). Incorporation of these known probiotic strains (which confer protection to IBD and colitis) in FMT therapy will eliminate risks associated with transfer of foreign unknown materials of the microbiota from fecal matter of donors. The ability for creating such probiotic incorporated therapies requires a detailed understanding of the *in vivo* mechanism of action of probiotic strains and disease pathogenesis. Strengthening the intestinal barrier using FMT can protect host from toxins released during CDI and other colonic infections. Microorganisms derived from ethnic dairy products can be a source of novel beneficial microorganisms which can limit unusual inflammatory responses and metabolic disorders (Bakken, 2014; Spinler et al., 2016).

Cardiovascular diseases are cause of serious threat to human life, as more than 17 million people died from these diseases in 2015 (Hajar, 2016). Different epidemiological studies carried out in last two decades have confirmed the correlation between cardiovascular diseases and total cholesterol (TC). In a study conducted on fermented dairy products like kefir, yoghurt, and cultured milk prepared from different milk sources, reduced cholesterol level was found compared to only milk (Bonczar et al., 2016). Cholesterol-lowering properties of only a few ethnic dairy products have been validated in animal models. A number of mechanisms have been put forward underlying the ability of probiotic bacteria to remove the cholesterol. These include hydrolysis of conjugated bile acid, assimilation of cholesterol, and precipitation of cholesterol along with bile salts. A study conducted on traditional Iranian dairy products (made of ewe milk) showed that both dead and live *L. brevis* could assimilate cholesterol (Iranmanesh et al., 2014). Another study conducted on *L. plantarum* showed similar anti-cholesteremic effect as shown by *L. brevis* in Iranian dairy product. Scanning electron microscopic (SEM) images in the study depicted that cholesterol gets adhered to the cell surface by both enzymatic assimilation and cell surface binding. (Choi and Chang, 2015).

A number of studies have indicated the role of kefir grains in cholesterol reduction. *L. plantarum* MA2 isolated from kefir has also shown hypocholesterolemic activity in male Sprague–Dawley (SD) rats fed with high cholesterol diet. Huang et al. (2013) observed that probiotic *L. plantarum* Lp27 isolated from Tibetan kefir grains was able to decrease serum total cholesterol, LDL-cholesterol, and triglycerides in hypercholesterolemic SD rats that consumed a diet supplemented with *Lactobacillus*. They further found that the Lp27 strain was able to reduce cholesterol absorption in Caco-2 cells by down-regulating the expression of Niemann-PickC1-like 1 (NPC1L1) in Caco-2 cells (Huang et al., 2013).

In another study carried out in SD rats fed with high cholesterol-containing diet, Zheng et al. (2013) observed that three strains, *L. kefiri* D17, *L. plantarum* B23, and *L. acidophilus* LA15, were able to lower the serum total cholesterol, LDL, and triglyceride levels as well (Zheng et al., 2013). In a similar *in vivo* study, milk fermented with *Lactococcus lactis* subsp. *lactis* IS-10285 was found responsible for reducing LDL cholesterol, total serum cholesterol, and total bile acids (Pato et al., 2004). In a recent clinical study on hypertensive overweight women, the impacts of *Lactobacillus casei* 01 (concentration-10^8^ cfu/g) when incorporated in Minas Frescal cheese was studied at pilot scale. The clinical study revealed that *L. casei* incorporation improved the low- and high-density lipoprotein cholesterol, total cholesterol, triacylglycerides, hematocrit, and hemoglobin count (Sperry et al., 2018).

In a recent study, 115 cultures isolated from ethnic fermented Tibetan yak milk were used to screen the cholesterol reduction ability. *L. plantarum* Lp3 was found to reduce cholesterol by 73.3% when administrated in rats fed with cholesterol-rich diet. A significant decline in liver and serum cholesterol was also detected (Ding et al., 2017). A study on yak milk fermented product concluded that the *Lactobacillus* isolated from ethnic yak milk has higher cholesterol reduction ability in comparison to other dairy products (Pan et al., 2011). The study showed that the mechanism behind the lowering effect of Lp3 was by assimilation and removal of cholesterol in feces due to deconjugation of bile acids.

A study using commercial and traditional kefir showed that there was a lower plasma cholesterol level when mouse models were fed with traditional kefir; however, when fed with commercial kefir, there was no such significant lowering of cholesterol. The study showed the beneficial role of traditional fermented milk product in lowering cholesterol level (Bourrie et al., 2018). A similar study conducted in Turkey on *L. fermentum* strains, isolated from Tulum cheese, showed that *L. fermentum* strains differ in their ability to assimilate cholesterol from media. It was observed that cholesterol assimilation in these strains ranged from 20.7 to 71.1% in media with bile. The authors also found that adhesion rates of some of these strains onto the Caco-2 cells were higher than that of control probiotic strain *L. rhamnosus* GG (Tulumoğlu et al., 2014). Furthermore, to find out whether traditional fermented food or traditional milk products serve as a better source of probiotic *Lactobacillus*, a comparative study was conducted on traditional homemade fermented cabbage called Suan-tsai and Koumiss. It was observed that there is a difference in cholesterol reduction ability of isolated probiotics from these sources. *Lactobacillus* isolated from Koumiss exhibited a higher cholesterol removal and bile tolerance ability than that of Suan-tsai samples. Thus, the traditional fermented dairy product can be a better source of potential probiotics when dealing with cholesterol assimilation ability (Guo et al., 2015). It can, therefore, be concluded that cholesterol reduction ability varies among different strains of lactic acid bacteria and ethnic foods can be potent source of these LAB.

The gut microbiota is comprised of resident commensal bacteria and transient probiotic bacteria which are consumed by fermented milk products (FMPs) and healthy foods. A correlation between gut microbiota and native inhabitants of FMPs has been observed in many studies. Veiga and coworkers, in a metagenomic study to observe the effect of FMPs on gut health, revealed that FMPs could substantially increase native beneficial bacteria like *Bifidobacteria* spp, along with the strains present in FMPs like *Streptococcus thermophilus* CNCM I-1630, *Lactobacillus delbrueckii* subsp*. bulgaricus* CNCM I-1632, *Lactobacillus delbrueckii* subsp*. bulgaricus* CNCMI-1519, and *Lactococcus lactis* CNCM I-1631. Three non-commensal species *Parabacteroides distasonis*, *Bilophila wadsworthia*, and *Clostridium* sp. HGF-2 were observed to be mitigated. FMPs also increased the native butyrate producers like *Roseburia intestinalis*, *Roseburia inulinivorans*, *Butyriovibrio crossotus*, *Clostridium* L2–50, *Faecalibacterium prausnitzii*, *Eubacterium hallii*, *Lachnospiraceae bacterium* 5-1-63FAA, *Coprococcus* ART55/1 and *Acidaminococcus*, *Bifidobactericae*, and *Firmicutes* (Veiga et al., 2014). Another study by Volokh and coworkers found significant augmentation of *Bifidobacterium* and *Firmicutes*, *Streptococcus isothermophilus*, and *Lactobacillus delbrueckii* after 30 days of FMP feeding. *Slackia isoflavoniconvertens* and *Adlercreutzia equolifaciens* were found to be increased with specific ability to metabolize isoflavone to equol, suggesting potential for multi-faceted positive impact of FMP (Volokh et al., 2017). Indian population belonging to the tribal parts exhibits higher consumption of FMPs than those consuming westernized foods, which may have increased beneficial gut microbiota. In a study, Mojibur R. Khan and coscientists have analyzed the gut bacterial profile of Mongoloid and Proto-Australoid tribes of India, where prevalence of *Prevotellaceae, Ruminococcaceae, Eubacteriaceae, Lachnospiraceae, Clostridiaceae, Veillonellaceae, Bacteroidaceae, Bifidobacteriaceae, Erysipelotrichaceae, Lactobacillaceae*, and *Coriobacteriaceae* have been observed. Among these strains, *Bifidobacteriaceae, Lactobacillaceae, Veillonellaceae, Clostridiaceae*, and *Eubacteriaceae* showed significant differences in their abundance across the population (Dehingia et al., 2015). Another study conducted using 1,000 subjects across India in a project called “Landscape Of Gut Microbiome - Pan-India (LogMPIE)” showed dominant bacteria belonging to the phyla of *Firmicutes, Bacteroidetes and Proteobacteria*. Rarer factions belonging to the phyla of *Verrucomicrobia* and *Spirochaetes* have been observed. This study also reports prevalence of *Prevotella* dominance in Indian cohorts (Shetty, 2018).

## Anticancer Attributes of Fermented Milk Products and its Associated Microbiota

**Figure 2:**
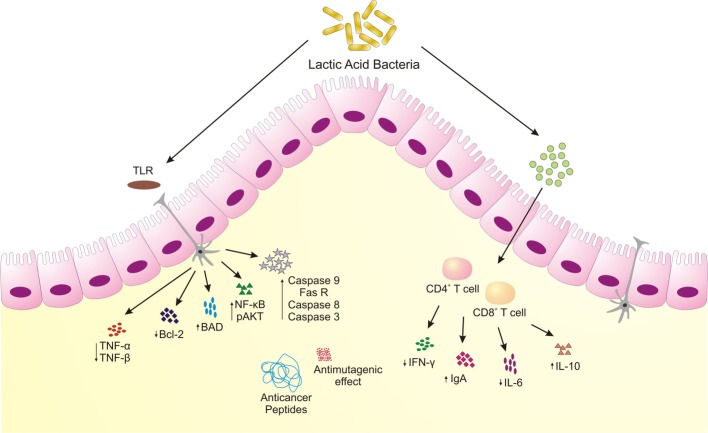
Schematic representing anticancer attributes of microorganisms associated with traditional fermented milk foods. LAB induce apoptosis, increased anti-inflammatory cytokines, produce bioactive peptides to restrain colonic and colon-associated cancer.

Alteration or disturbances in gut milieu has an apparent effect on cancer development. Native gut inhabitants possess negative effect on cancer metabolism and prognosis (Zitvogel et al., 2017). Diverse mechanisms attribute to the anticancer properties of probiotics, prebiotics, and synbiotics (Figure 2). These include inhibition of mutagen- and carcinogen-producing microbes, protection from oxidative stress, metabolism of carcinogen and xenobiotics, immunomodulation, and altering expression of different genes in cancer metabolism like metastasis, cell cycle control, apoptosis and cell death, cancer stem cell inhibition, modulation of intestinal microflora, and inhibition of tyrosine kinase pathway (Raman et al., 2013; Liévin-Le Moal and Servin, 2014). It has been seen that probiotic bacteria have modulatory and anti-proliferative effect on different cells and cell types. Probiotics have been shown to affect different stages of metastasis like cellular adhesion, invasion and intravasation, maintenance of tumor microenvironment, and cancer stem cell homeostasis. Cell-to-cell adhesion plays a critical role in protecting cellular integrity of the tissues and any flaw in the system augments the metastasis process. Probiotics affect different stages of metastasis like cellular adhesion, invasion and intravasation, and maintenance of tumor microenvironment and cancer stem cell homeostasis (Motevaseli et al., 2017). The mechanisms span a wide range of lactic acid bacteria isolated from individual and indigenous ethnic milk fermented products. Although, the anticancer potential of extant human microbiota is being thoroughly investigated, yet, exploring mechanistic insights of anticancer activity of indigenous fermented milk products and their inhabiting microbiota is of paramount importance.

Several studies have been conducted on anti-proliferative effect of kefir. Kefir is a popular, indigenous fermented milk product of West Asia, comprised of a diverse group of lactic acid bacteria and yeast. Both kefir and cell-free extract of kefir could regulate expression of various genes involved in apoptosis and survivability in Caco-2 and HT-29 cancer cells. Kefir could arrest cell cycle in G2 phase and induce cell death. Using real-time and western blot studies, the authors showed that kefir could successfully decrease transforming growth factors (TNF-α and TNF-β) in HT-29 cell line which are essential for cell proliferation. Kefir also increases Bax-Bcl-2 ratio, which substantiates pro-apoptic effect of the extract. Interestingly, they observed no alteration in expression of matrix metalloproteinase and intravasation/mitigation of breast and colon cancer cells (Khoury et al., 2014). However, another study showed that cell-free extract of kefir displayed anti-metastasis effect on lung carcinoma and B16 melanoma cells (Furukawa et al., 2000). Metastasis does not only depend upon metabolites, but also on microenvironment, intravasation of vessels, and colonization of the secondary sites (Fidler, 2003). Chen et al. (2007) have demonstrated the effect of kefir in mammary cancer cells (MCF-7) and compared kefir with yoghurt extract. They found that kefir displayed anti-proliferative effect on cancer cell population upto 56%, whereas yoghurt could reduce only 14%. Capillary electrophoresis result showed that the content of peptide in kefir was much more than that of yogurt, which could be a probable reason for its enhanced anti-proliferative effect (Chen et al., 2007). Numerous studies have demonstrated anticancer effect of peptides (Deslouches and Di, 2017). *L. rhamnosus* GG, a bacterium isolated from human infant feces, expresses proteins p50 and p75, which are established to have anti-proliferative effect on cancer cells (Liévin-Le Moal and Servin, 2014). Kefir also delayed tumor development when fed to a breast cancer model of mouse for 2–7 days in a dose-dependent manner. Within 2 days, both the kefir and kefir-free extract increased IL-10 and reduced IL-6 expression in serum, which perturbs estrogen homeostasis in mammary gland. It was furthermore noticed that kefir could reduce TNF-α and INF-γ concentrations in breast cancer cells, factors required for cell proliferation. Kefir also augmented cellular apoptosis by reducing Bcl-2 protein in mammary glands. Involvement of peptides from the probiotic organisms and contents of the kefir was held as an important factor for tumor mitigation (Furukawa et al., 1990; Vinderola et al., 2005). Shiomi et al. (1982) observed that the molecules exerted by the fermentation of the bacteria displayed cytotoxicity to the cancer cells rather than the bacteria itself (Shiomi et al., 1982). Moreover, a number of probiotics that were isolated from fermented dairy products have also been found to have anti-*H. pylori* effects (Nair et al., 2016).

Dubey et al. (2016) showed promising effect of *P. pentosaceus* GS4 on colon cancer mitigation. *P. pentosaceus* GS4 strain was isolated from khadi, an Indian fermented dairy-based food. It showed anti-proliferation activity of HCT-16 mammalian cells, and increased expression of pro-apoptotic molecules NF-κB and p-Akt. In animal model, it decreased the severity of the cancer, augmented oxidative stress and necrosis (Dubey et al., 2016). The authors demonstrated the effect of conjugated linoleic acids on cancer mitigation. The gut mirobiota probably helps biohydrogenation to produce conjugated linoleic acid (CLA) which in turn triggers apoptosis, caspase activity, and cleavage of poly-ADP ribose polymerase (PARP). It was previously demonstrated that *P. pentosaceus* could increase Bax/Bcl-2 ratio which subsequently increases mitochondria-mediated membrane permeability and inhibits cancer cells. It has been seen that local delivery of CLA could modulate gut microenvironment in mitigation of colon cancer (Bassaganya-Riera et al., 2012; Dubey et al., 2015). Linoleic acid found in gut could be bio-transformed to CLA by intestinal microbiota which explains the pro-apoptotic and anti-proliferative effect (Edionwe and Kies, 2001).

Traditional Iranian dairy products like tarkineh, shiraz, yogurt, and cheese contain various species like *L. plantarum* and *L. lactis subsp*. lactis which showed anticancer activity against HT-29, AGS, MCF-7, and HeLa cells. The pro-apoptotic activity of the bacterial secreted products can be compared to the cytotoxic potential of taxol, an anticancer phytochemical. Haghshenas et al. (2015) demonstrated that the anti-proliferative effect of the fermented products reduced after pronase (a protease cocktail) treatment, which depicted proteinaceous nature of the bioactive metabolites (Haghshenas et al., 2015). *L. plantarum* strain is also a dominating lactic acid bacterial species found in ethnic dairy products like Armada cheese, Batzos cheese, yoghurt, Laban zeer, Kulenaoto, M’Bannick, Kwerionik, Koumiss, and Zincica (Psoni et al., 2003; Bernardeau et al., 2008). In another study, Haghshenas et al. (2015), while working with Iranian traditional yogurt, found two *Acetobacter* species, *Acetobacter indonesiensis* and *Acetobacter syzygii*, responsible for exhibiting cytotoxicity toward HeLa, MCF-7, AGS, and HT-29 cancer cells (Haghshenas et al., 2015). The secreted products of both the strains showed significant inhibition of cell proliferation without hindering the physiology of normal cells. Similar to the previous studies by the group, pronase treatment significantly reduced the effect of secreted products, due to the involvement active peptides or enzymes responsible for transformation of carcinogenic and xenobiotic compounds (De LeBlanc and Perdigón, 2010). The secreted metabolites also displayed pro-apoptotic behavior when treated with the above-mentioned cell lines, with observable physiological disorders like membrane blebbing, nucleus fragmentation, cell shrinkage, and apoptotic body formation (Haghshenas et al., 2015). Microbial samples isolated from yoghurt and cheese from rural areas of Kurdistan province of Iran contain highly beneficial yeast *K. marxianus* AS41. Saber et al. (2017) observed that *K. marxianus*-secreted metabolites significantly down-regulated Bcl-2 expression and up-regulated BAD and Caspase 9 gene expression in epithelial cancer cells, which subsequently increased apoptosis and reduced cell proliferation. In addition, Fas R, caspase 8, and caspase 3 were also observed to be up-regulated, the factors involved in intrinsic apoptotic pathway. The secreted metabolites demonstrated the apoptotic effect on cellular physiology by DNA fragmentation, chromatic condensation, and membrane blebbing in HT-29, Caco-2, and Hep-2 and Hep-G2 cells (Saber et al., 2017).

De LeBlanc et al. (2005) showed that *Lactobacillus helveticus* R389 (a strain isolated from Swiss Cheese) when fed to a mouse with breast cancer, increased IgA and CD4 positive cells 4 days post injection. The strain also showed decreased IL-6 and increased IL-10 expression. The fermented milk of *L. helveticus* R389 increased the IgA positive cells, eventually stimulated immune system, and inhibited growth of immune-dependent fibrosarcosoma in mouse model. *L. helveticus* also delayed the growth of breast cancer in balb/c mouse. It was inferred that the metabolites possibly modulated endocrine system, as decreased IL-6 expression repressed estrogen-dependent tumor development (De LeBlanc et al., 2005). *L. helveticus* also exerted anti-mutagenic effect in Ames test, where milk fermented by the proteolytic strain could inhibit mutagenesis significantly (Matar et al., 1997).

Camel milk fermented products like Chal and Shubat are very important part of daily cuisine in North Africa and mid-western Asia. Still, limited literature is available on mechanistic insight of anticancer properties of fermented camel milk. In a recent study, anticancer property of water-soluble extracts (<3 KDa) of fermented bovine and camel milk was evaluated against MCF-7, Caco-2, and HeLa carcinoma cells. It was observed that the proliferation of MCF-7, Caco-2, and HeLa cells was significantly inhibited by water-soluble extracts of camel milk rather than bovine milk fermented by strains like *L. plantarum*, *L. acidophilus*, *L. reuteri*, and *L. lactis*. The authors concluded that the high anti-proliferation activity of fermented camel milk prepared using these strains may have contributed to the difference in peptides derived from fermented camel milk rather than those from fermented bovine milk. They correlated proliferation inhibition with angiotensin-converting-enzyme (ACE)-inhibitors, which suggested that peptides derived from fermented camel milk have multifunctional bioactivity (Ayyash et al., 2018).

Anti-mutagenic effect of probiotic metabolites is also of paramount importance for prevention of cancer. Ahmadi et al. studied 25 bacterial strains isolated from the Tarhana, an indigenous Turkish food, based on grain and yoghurt or fermented milk, a similar dish like Kishk. Species of *L. brevis*, *L. plantarum*, and *L. casei* isolated from Tarhana showed high anti-mutagenic and anticancerous effect (Abbas Ahmadi et al., 2014). In a study conducted on strains isolated from Dadih, an Indonesian ethnic fermented milk of West Sumatra, *Enterococcus faecium* IS-27526 was found to lower down the fecal mutagenicity in rats as compared to the milk cultured with *L. plantarum IS-20506* (Surono et al., 2009).

Recently, Navani et al. (2018) filed a patent on asparaginase produced by *L. brevis* isolated from Himalayan yak cheese Chhurpi. Asparaginase is an anticancer enzyme which depletes free L-asparagine in blood, leading to death of leukemic cells resulting from starvation (Navani et al., Patent no. 201811019299/New Delhi, India; unpublished data). Proteins like asparaginase may hold a key to explore more metabolites associated with anticancer attributes. Previously, Haghshenas and coworkers have discussed the role of anticancerous peptides and proteins in traditional fermented milks, and proteins like asparaginase may have a role in their study (Haghshenas et al., 2015). Probably enzyme like asparaginase in combination with peptides and small metabolites exert synergistic effect toward anticancer activities of fermented dairy products.

The beneficial effect of probiotic dairy products and their extant microbes in cancer are being studied thoroughly throughout the world and found to be implicated in several pathways of cancer metabolism, angiogenesis, and metastasis. The bacteria or their bioactive molecules alter the expression of different genes and restrain varied pathways in cancer progression. Studies on the strain-specific bioactive compounds, immunoregulation, time-dependent transcriptomics, and metabolic studies will guide a better picture toward the application of the fermented products and their inhabitant microbes. Furthermore, randomized clinical studies should be conducted to translate the observations for medical use.

## Beneficial Perspective of Bioactive Metabolites Present in Ethnic Fermented Dairy Products

**Figure 3:**
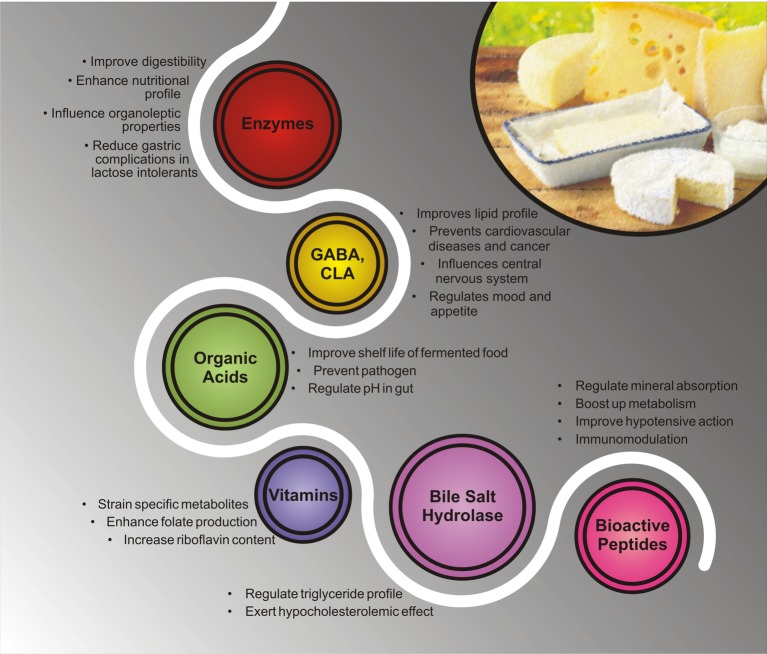
Different health benefits of bioactive metabolites present in ethnic fermented dairy products.

During fermentation, microorganisms metabolize complex food matrix and synthesize bioavailable and bioactive compounds leading to healthy consequences for humans (Figure 3). In dairy industry, LAB are widely used as starter culture as they play crucial role in fermentation. At industrial scale, LAB are used for the synthesis of numerous primary or secondary metabolites like enzymes, organic acids, and vitamins (Patel et al., 2013). Microflora (*L. mesenteroides*, *L. lactis,* and *L. paracasei*) present in Kajmak (a fermented dairy product of soft and creamy texture found in mid-western Asia and Eastern Europe) contribute in developing aroma and flavor of the product by producing diacetyl, acetate, and ethanol (Jokovic et al., 2008). Acetate acidifies its surrounding environment, resulting in inhibition of pathogenic bacteria. Acetate and propionate control sugar metabolism *in vivo* by alleviating glycaemia and improving insulin sensitivity (Turnbaugh et al., 2006). *L. acidophilus, Streptococcus cremoris*, and *Streptococcus lactis* majorly found in camel’s milk and Chal are used as starters in dairy products. Wide varieties of lactic acid bacteria present in camel’s fermented milk assimilate carbohydrates like galactose, mannose, lactose, and xylose. Dairy yeast *K. marxianus* and *K. lactis* also carry ability to assimilate sugars like lactose and galactose (Yam et al., 2015; Saini et al., 2017b). *L. plantarum* RYPR1, an indigenous probiotic strain isolated from Indian fermented beverage Raabadi, showed hypocholesterolemic property due to bile salt hydrolase activity. Antibacterial activity of isolated strain was also observed against tested pathogens like *E. coli*, *S. aureus*, *P. aeruginosa,* and *S. albony* (Yadav et al., 2016).

Enzymatic activity of microflora present in fermented food contributes to improved digestibility and nutritional value of food. In other words, beneficial microorganisms present in fermented food may be used for the synthesis of enzyme extracts which are stable under native environment of fermentation and transform complex food matters into simpler ones (Tamang et al., 2016a,b,c). For example, thermophilic strain *L. delbrueckii* subsp. *bulgaricus* and *S. thermophilus* used for the production of yogurt contain substantial amount of β-D-galactosidase enzyme which has been found to improve lactose malabsorption in people suffering from lactose intolerance (Tamang et al., 2016a,b,c). Many strains of LAB isolated from different fermented products produce also antimicrobial compounds such as bacteriocin by *L. lactis* and pediocin by *P. pentosaceus* (Tamang et al., 2016a,b,c). Likewise, cheese, a fermented dairy food is also consumed by lactose-intolerant people as some amount of lactose present in the milk is fermented and the rest is processed into the whey at the time of cheese manufacturing (Kokkiligadda et al., 2016; Marco et al., 2017). Enzymatic activities of LAB have been extensively studied and were found to produce diverse enzymes which affect food quality, texture, and organoleptic attributes. Proteolytic and lipolytic activity of LAB improves sensory quality of cheese. *Lactococcus lactis* subsp. *cremoris* produces peptidases which improve flavor and aroma of cheese (Guldfeldt et al., 2001, González et al., 2010). Enzymatic activity of LAB strains—*Lactococcus* sp. and *Enterococcus* sp.—associated with Spanish cheese Genestoso showed high activity of dipeptidase, leading to their enterolytic activity (González et al., 2010). A study showed that strains belonging to the genera *Lactobacillus* produce hydrolytic enzymes in gastrointestinal tract which positively influenced digestive process and reduced probability of malabsorption (Naidu et al., 1999). Fermented foods also harbor *Lactobacillus*, *Lactococcus*, *Bifidobacterium*, and *Pediococcus* which synthesized enzymes responsible for carbohydrate degradation such as xylanases, glucosidases, and amylases (Novik et al., 2007; Patel et al., 2012).

Conjugated linoleic acid (CLA) has numerous beneficial effects on many health-related complications such as inflammatory diseases, cancer, metabolic issues, and cardiovascular diseases. LAB inherent in fermented dairy products enriches the product with increasing content of CLA. One study corroborating the fact showed that *L. lactis* subsp. *cremoris* MRS47 from Brazilian kefir grains has ability to modulate lipid profile (increased SCFA) of milk by fermentation. During fermentation, elevated amount of polyunsaturated fatty acid and reduced saturated fatty acid content were also observed (Vieira et al., 2017).

LAB produce amino acid derivatives and oligopeptides because of their proteolytic activity in fermented food. These bioactive molecules play important role in regulating many vital activities such as mineral absorption, metabolism, cardiovascular activities, immune modulations, and mood alteration (Pessione and Cirrincione, 2016). Proteolytic system of LAB produces many bioactive peptides from milk protein, especially from casein and whey protein. It was found that casein hydrolysis-generated peptides influenced gut-brain axis activities, cardiovascular functions, antimicrobial activity, nutrition, and immunomodulatory attributes (Thakur et al., 2012). *L. helveticus* CRL 1062, a common constituent of lactic starter culture used in cheese industry, hydrolyzed α- and β-caseins through cell envelop proteinase (Hébert et al., 1999). *L. helveticus* CRL 581, isolated from cheese, hydrolyzed β-casein more quickly in comparison to α-casein due to proteinase enzyme associated with the cell membrane (Hébert et al., 1999). Bioactive peptides released from hydrolysis of milk whey proteins (alpha-lacto-albumin, beta-lacto-globulin, lactoferrin, and immunoglobulins) produced bioactive peptides, had hypocholesterolemic property which can minimize absorption, and increased excretion of cholesterol in feces (Nagaoka et al., 2001).

ACE plays an imperative role in increasing blood pressure by generation of vasoconstrictor angiotensin II (a component of renin-angiotensin system) and down-regulating the expression of vasodilator bradykinin. Drugs to restrain ACE in blood are commonly used in hypertension, myocardial infarction (heart attack), and diabetes (Ma et al., 2010). Renin-angiotensin-aldosterone is the primary blood pressure controlling system. ACE inhibitory peptides casokinins and lactokinins are produced by proteolytic digestion of casein (FitzGerald and Meisel, 2000; FitzGerald et al., 2004). Blocking the generation of Angiotensin II, the peptides lower arterial resistance, increase intravenous capacity, decrease cardiac output and index, and lowered resistance in blood vessels in kidneys and increased excretion of sodium and urine. Dairy isolates *Lactobacillus delbrueckii* subsp. *bulgaricus* SS1 and *L. lactis* subsp. *cremoris* FT4 are responsible for the synthesis of ACE inhibitor peptides in fermented dairy products. In another study, homemade Argentinian hard cheese-isolated strain *Lactobacillus delbrueckii* subsp. *lactis* CRL 581 was investigated for its cell envelope-associated proteinase (CEP) activity. It was found that *Lactobacillus delbrueckii* subsp. *lactis* CEP was able to hydrolyze both α- and β-casein except k-casein and produced antihypertensive peptides (Hebert et al., 2008). Generally, LAB peptidases enhance ACE inhibitor activity by minimizing the chain composed of poly/oligopeptide. Oligopeptides having antihypertensive property, derived from casein, are 2–6 amino acids long (Pessione and Cirrincione, 2016). *L. helveticus* PR4, obtained from Italian cheese, produced ACE inhibitory and antibacterial peptides by hydrolyzing casein milk protein. Antibacterial peptides showed wide range of inhibition against tested pathogens *Enterococcus faecium*, *Bacillus megaterium*, *Escherichia coli*, *Salmonella* spp., *Yersinia enterocolitica*, and *Staphylococcus aureus* (Minervini et al., 2003).

Some LAB species can decarboxylate amino acids such as gamma-amino-butyrate (GABA) which is a decarboxylated product of glutamate. Likewise histamine, β-phenylethylamine and tyramine are decrboxylated products of histidine, phenylalanine, and tyrosine respectively (Konings, 2006). These amines such as histamine, tyramine, tryptamine, beta-phenylethyl amine, and GABA have beneficial impact on vascular or central nervous system of humans (Moreno-Arribas et al., 2003). GABA and beta-phenylethyl amine are responsible for relaxing gut smooth muscles, controlling appetite as well as the mood. *L. brevis* PM17, *L. plantarum* C48, *L*. *paracasei* PF6, *L. delbrueckii* subsp. *bulgaricus* PR1, and *L. lactis* PU1 were reported as the maximum producer of GABA in comparison to other isolated species from Italian cheese (Siragusa et al., 2007). In another study, 81 strains of *Lactobacillus* were isolated from the Koumiss procured from Xinjiang, China, and screened for ACE inhibitory activity and GABA production. Koumiss is traditional fermented milk made of mare’s or camel’s milk. It was found that 16 strains showed ACE inhibitory activity, out of which two strains showed significant GABA-producing ability. *Lactobacillus helveticus* ND01 showed good ACE inhibitory and high GABA synthesis capability as well (Sun et al., 2009).

Several metabolites produced during fermentation are strain specific. LAB produce vitamins (e.g. folate) in variety of fermented dairy products such as curd, yoghurt, cultured butter milk, cheeses etc. Amount of folate in yoghurt depends on starter cultures (Wouters et al., 2002). Folate is a water-soluble vitamin B and very important for human health. Deficiency of folate may lead to a variety of health complications like osteoporosis, Alzheimer’s disease, coronary heart disease, and high risk of breast and colorectal cancer (Rossi et al., 2011). Folate biosynthesis is strain-dependent property, as many *Lactobacillus* spp. and *Lactococci* spp. Like *L. plantarum, L. bulgaricus, L. lactis*, *S. thermophilus*, and *Enterococcus* spp. are able to produce folate while some lactobacilli (*L. gasseri, L. salivarius, L. acidophilus*, and *L. johnsonii*) cannot due to absence of genes responsible for folate biosynthesis (LeBlanc et al., 2007). In a study, cow’s milk-isolated folate-producing lactic acid bacteria were screened for probiotic properties. It was found that *L. lactis* subsp. *cremoris* and *L. lactis* subsp. *lactis* showed efficient probiotic properties with significant folate production (Gangadharan et al., 2010). These folate-producing strains can be used for developing fermented dairy food with good nutrition profile. Riboflavin, a precursor of the coenzymes flavin mononucleotide (FMN) and flavin adenine dinucleotide (FAD) have been shown to be produced by *L. acidophilus* isolated from yoghurt samples in Vellore, India (Jayashree et al., 2010). Whey was recommended as a better fermentation medium compared with skim milk for riboflavin production (Guru and Viswanathan, 2013). The gut bacteria can also transform anti-nutritive factors present in cereals or plant products into nutritional metabolites improving nutritional value of the food product. Phytase-producing strains have long been used in degradation of phytate in wheat dough to increase calcium, phosphorus, and magnesium in food (Lopez et al., 1983, 2000; Reale et al., 2004; Palacios et al., 2008). Sharma et al. (2018) have recently reported a novel tyrosine phosphate like phytase from a probiotic bacterium *L. fermentum* NKN51 isolated from Himalayan yak cheese Chhurpi. The enzyme showed high specificity to its substrate phytate, a compound which chelates micronutrients and cationic proteins and limits their availability in food. Phytase from *L. fermentum* NKN51 also showed significant dephytanation of finger millet and Durum wheat under *in vitro* gastrointestinal conditions and displayed potential as food and feed additive (Sharma et al., 2018). Novel phytase have also been reported in other probiotic strains like *Bifidobacterium pseudocatenulatum* (Tamayo-Ramos, 2012), and *Lactobacillus sanfranciscensis* (De Angelis et al., 2003).

Probiotic strains improve the nutritional profile of fermented food by introducing their metabolites in food matrix. Bioactive metabolites constitute functional peptides, small molecules, short-chain fatty acids, vasodilators, vitamins, immunoregulators, and numerous other factors facilitate healthy and proper vital activities in human body. Thus, incorporating fermented food in daily eating habit can be a smart approach to attain healthy living.

## Recent Advancements in Gut Microbiome Research

Recent advances in Next Generation Sequencing (NGS) approaches have completely altered gut microbiome research. “Omics” studies have facilitated researchers to explore *in situ* microbiome both temporally and spatially, complex microbial communities targeted phenotype studies to explore potential role in human health. Nicola Segata and coworkers have provided the largest ever catalog of human-associated microbes across the world population. They analyzed bacterial genomes from oral cavity, skin, vagina, and stool samples of 32 countries taking westernized and non-westernized lifestyle and also included cohorts from Madagascar. The metagenomic samples of nearly 9,500 individuals reconstituted 154,723 new microbial genomes from 5,000 species. Around 77% is found to be unexplored till date. Many of the beneficial bacteria like *Succinatimonas* spp., *Bifidobacteria* spp., and *Firmicutes* spp. were found to be prevalent in non-westernized communities suggesting host lifestyle relating to healthier food habits. Whereas, bacteroids were majorly found in westernized factions. The study has reflected on the diversity of bacteria related to antibiotics, complex industrialization, and unhealthy lifestyle (Pasolli et al., 2019). A study on the presence of lactic acid bacteria in ethnic fermented milk of North-Eastern India by Tamang and coworkers depicts presence of Firmicutes (*Streptococcaceae, Lactobacillaceae*) and Proteobacteria (*Acetobacteraceae*) as the predominant species, *L. lactis* and *L. helveticus* being major lactic acid bacteria and *Acetobacter* spp. and *Gluconobacter* spp. as the chief acetic acid bacteria present in these products (Shangpliang et al., 2018). Moreover, recent NGS studies on cheese from pasteurized and unpasteurized milk showed difference in bacterial compositions relating to ripening, aging, coloration, and beneficial role of probiotic organisms (Salazar et al., 2018). The discovery of bacterial populations in culture-independent method has rejuvenated the significance of microbial ecology in fermented food and important functionality of the products.

Studies with large cohorts have shown pivotal role of early life microbiota in obesity and type I diabetes (T1D). Researchers identified biomarkers for assessing the role of these organisms later in adulthood. These findings depict that genes related to various functions like fermentation and biosynthesis of SCFA have more relevance than particular taxa in prognosticating development in metabolic and autoimmune diseases. Moreover, the study also conveyed that attention has to be paid in developing the patterns of beneficial microbial communities in childhood for infant health (Stanislawski et al., 2018, Stewart et al., 2018, Vatanen et al., 2018). World Gastroenterology Organization has recently launched new guidelines for physicians with science-based formulations to improve gut health *via* diet. Scientists have started exploring the effect of macronutrients like omega-3 polyunsaturated fatty acids, micronutrients, and food additives in broadening the dietary patterns. Food groups are recommending diet with different fibers, based on fermentability rather than solubility. Overall, scientists provided guidelines around various fibers and macronutrients affecting the gut health instead of isolated nutrients for thriving individual taxa of microbe (Halmos et al., 2015; Staudacher et al., 2017; Makharia et al., 2018). Researchers have elucidated the role of non-antibiotic drugs like proton-pump inhibitors, antipsychotics, and metformin in altering gut microbiota, which may have role in gastrointestinal side effects, therapeutic actions, and antibiotic resistance. In a study by Maier and coworkers, it was perceived that out of 835 human-targeted drugs, only a few strains were affected by these drugs; however, 40 drugs affected more than 10 strains. Species affected were *Eubacterium rectale, Roseburia intestinalis, Coprococcus, Bacteroides vulgatus*, *Prevotella copri*, *Blautia obeum*, whereas gamma-proteobacteria were almost resistant to those drugs (Maier et al., 2018). New evidence in host–microbe interaction studies revealed new mechanistic insights into role of *Lactobacillus rhamnosus* CNCM I-3690 in maturation of intestinal barrier’s structure and functionality in mice (Natividad et al., 2013; Tlaskalova-Hogenova et al., 2015; Hayes et al., 2018). One of the major findings in recent years showed that significant source of antibiotic resistance genes in infant gut microbiota was from mother’s gut and breast milk. Scientists have developed new methods for determining genes conferring resistance in gut microbiota and role of these genes in bacterial pathogenesis (Pärnänen et al., 2018). These show concern in propagation of antibiotic resistance and multiple antibiotic-resistant bacteria in infants. New studies have also shown that probiotics can regulate the need of antibiotics from childhood, which subsequently can mitigate the rise of antibiotic resistance. The studies showed that probiotics supplementation is more effective than placebo for reducing certain illnesses, which ultimately reduces the use of antibiotics (King et al., 2018). The past decade has seen a huge development in understanding microbiota gut-brain axis and its importance in ailment of neurodegenerative disorders. Recently, with the discovery of “neuropod cells,” scientists have displayed how the gut lumen communicates rapidly with the brain after meal and role of microbiota in regulating homeostasis and proper signaling (Kaelberer et al., 2018).

## Conclusion and Future Perspectives

The near explosion in the knowledge about gut microbiome and their role in metabolism has advanced our understanding about how intimately human health is related to microbes. Metabolism has direct relation with homeostasis of body functions as microbiota related to food and beverages play a pivotal role in modulation of host defense, host–microbe interaction, and epigenetic changes. As fermented indigenous foods have been serving communities since the dawn of civilization, microbiota associated with these foods is interconnected with healthy and safe attributes. The beneficial effect of these indigenous foods and associated microbiota includes up-regulation of immune system, strengthening gut-brain barrier, regulation of immune modulators, mitigation of carcinogens, induction of apoptotic pathways, and production of numerous metabolites. Diseases like IBD, colitis, IBS, lactose intolerance, peptic ulcers, vaginosis, and hypercholesterolemia can be treated successfully with probiotics. New evidences suggest that these microorganisms also help in improving brain functions, alleviate age-related diseases, and reduce hazardous metabolites from the body.

For optimized and safe utilization of these microbes for human welfare, deeper understandings of mechanistic details of their functional attributes with controlled clinical trials are required. It will also be important to know microbial interactions and understand the molecular mechanism of such interactions so that such properties can lead to designer fermented products with predefined health attributes. Furthermore, comprehensive knowledge of metabolites and regulatory networks of these microorganisms could provide a platform for mimicking natural modulations and implementing them in treating disorders. Genetic manipulations and strain improvement will certainly enhance the valuable attributes. It is imperative for the food and dairy industries to study these indigenous dairy products and improve on prolonging shelf life, better adhesion, and survival in intestine for desired benefits and production for the global consumers. In addition, archiving microbiota from indigenous foods is also important as it will provide information about the attributes of starter cultures for improvement of aroma, texture, and flavor in dairy foods for posterity.

## Data Availability

All datasets generated for this study are included in the manuscript.

## Author Contributions

TG, AB, and AS participated in all steps of preparation of this manuscript. AB and NN participated in the editing of the manuscript and revised it critically.

### Conflict of Interest Statement

The authors declare that the research was conducted in the absence of any commercial or financial relationships that could be construed as a potential conflict of interest.
